# Feasibility of embedding orthopaedic clinical trials into national registries: a pilot quality improvement study for the UK Non-Arthroplasty Hip Registry (UK-NAHR)

**DOI:** 10.1093/jhps/hnae018

**Published:** 2024-06-13

**Authors:** Mark Andrew Sohatee, Callum McBryde, Tony Andrade, Paul Gaston, Jonathan Hutt, Vikas Khanduja, Ajay Malviya

**Affiliations:** Royal Derby Hospital, University Hospitals Derby and Burton NHS Foundation Trust, Derby DE22 3NE, England; The Royal Orthopaedic Hospital NHS Foundation Trust, Birmingham B31 2AP, England; Royal Berkshire NHS Foundation Trust, Reading RG1 5AN, England; NHS Lothian, Royal Infirmary of Edinburgh, Edinburgh EH16 4SA, Scotland; University College London Hospitals NHS Foundation Trust, London NW1 2PB, England; NHS Foundation Trust Cambridge, Addenbrooke’s Hospital, Cambridge University Hospitals, CB2 0QQ England; Northumbria NHS Foundation Trust, Newcastle upon Tyne NE63 9JJ, England

## Abstract

The integration of ‘Registry-based Randomised Control Trials’ (RRCT) into national registries has the potential to catalyse prospective research, enhancing the evidence base for practice. The aim of this study was to assess the feasibility of embedding a trial within the UK Non-Arthroplasty Hip Registry. This was a national observational, multi-centre study. Six pilot sites within the UK were provided with additional support for data collection. We compared the ability of these pilot sites to collect data with the ability of centres where no additional support was provided. We collected information on patient compliance, efficacy and adverse events of drugs routinely used after hip preserving surgery. The primary outcome measure was compliance with data collection in these centres at 30 and 90 days after surgery. Our intention was to assess the feasibility of, and factors influencing, the capturing data for interventional registry trials in the future. Two hundred and twenty-eight patients were enrolled in the Non-Arthroplasty Hip Registry during the study period (114 within pilot centres and 114 in non-pilot centres). Pilot centres had a mean follow-up compliance of 79% (30 days) and 69.4% (90 days) in contrast to 55% (30 days) and 47% (90 days) in the non-pilot centres (*P* = 0.009/*P* = 0.0058). The study revealed that supplementary administrative support resulted in improved compliance. However, deficient administration systems negatively impacted follow-up, and surgeon motivation emerged as a crucial determinant in ensuring robust follow-up. The lessons learned from this feasibility trial could be useful for any national registry embedding prospective, registry-based trials.

## INTRODUCTION

Publications from orthopaedic registries have allowed clinicians to analyse ‘big data’ [1], looking at both clinical [2–5] and patient-reported outcome scores [6–8] after surgery and have been valuable in assessing the outcomes in large groups of patients. However, registry studies are retrospective analyses of prospectively collected data and, therefore, fall short of large prospective and interventional studies, when considering levels of evidence [9–11]. To the authors’ knowledge, a national registry has not been utilized within the UK to undertake a prospective trial; furthermore, there do not appear to be any prospective registry trials in orthopaedics from any other countries. However, a newly defined methodology of the ‘Registry-based Randomised Control Trial’ (RRCT), has discussed the role of using an existing registry to collect treatment and outcome data [12].

There is limited evidence that such a registry-based trial can be embedded into the UK National Health Service. A recent scoping review looked at registry-based trials [10], in this context randomized studies, and included studies from Netherlands, USA, Sweden, Denmark, Australia and Italy. The studies from these six countries were in the clinical areas of cardiology, immunization, oncology and critical care. Some of the perceived benefits of using registries to undertake prospective/randomized studies are the ability to recruit and identify patients more effectively [13, 14] and to also reduce the administrative burden associated with data collection. This should also, theoretically, have benefits from a cost perspective [10], given that the cost of conducting research with the help of trial units is often prohibitively expensive. However, one of the existing problems from registry-based studies, conducted retrospectively, is the loss of patients to follow-up [15–18].

Given that there have been no current UK-based nor, to the best of our knowledge at the time of this study, any orthopaedic-based prospective registry trial, the Non-Arthroplasty Hip Registry (NAHR) set out to undertake a feasibility study in the first instance to ascertain whether prospective studies could be undertaken within the UK-NAHR. This is considering the aforementioned loss to follow-up, which has previously had an impact on studies undertaken by the NAHR and other registries alike. Furthermore, from the aspect of quality improvement, we looked at whether providing funding at selected pilot centres would result in improvements in follow-up.

## MATERIALS AND METHODS

The UK-NAHR was set up in 2012 under the British Hip Society to collect the outcomes of the growing number of hip preservation (non-arthroplasty) procedures being performed in the UK. It aims to collect data on patients undergoing ‘non-arthroplasty hip surgery’, which involves surgery data and patient-reported outcome measures. It does this through a data collection tool called the ‘Minimum Data Set’ (MDS), and the whole process is digital, relying on the surgeons, or their team, completing the electronic, web-based form preoperatively and the patients responding to the automated emails from the data controller to complete the postoperative forms at various time points in their postoperative recovery. It currently has more than 16 000 procedures entered in the registry by more than 100 surgeons. It aims to improve patient outcomes and allows surgeons to benchmark their performance according to minimum standards. The NAHR has already been used to generate high-quality research presentations and publications from both the user group and individual surgeons [8, 19–21].

**Fig. 1.**
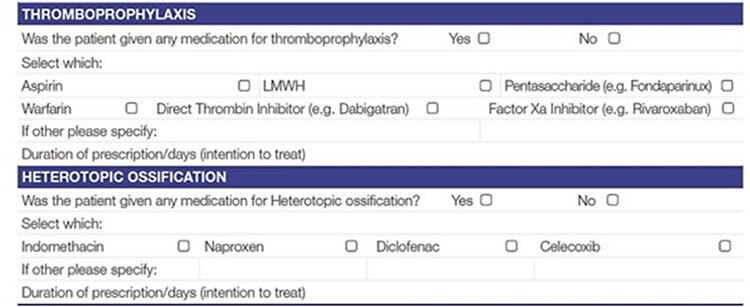
Amended section from the NAHR MDS tool for data capture.

In this study, we assessed the compliance of the patients to complete 30- and 90-day questionnaires [22] with regard to their use of certain medications that may be routinely prescribed after hip preservation surgery [8] [Fig. 1]. This was to ensure no deviation from routine practice. These were for thromboprophylaxis [6] and non-steroidal anti-inflammatory medications for heterotopic ossification due to its increased risk in hip arthroscopy [7].

**Fig. 2.**
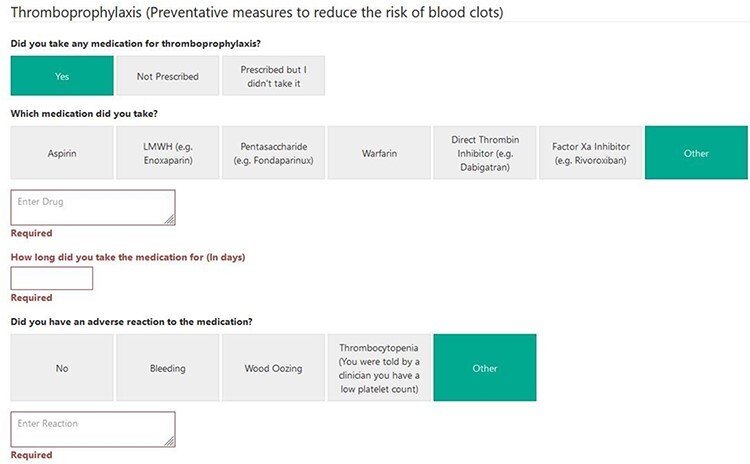
MDS tool for data capture at 30/90 days for Venous Thrombo-Embolism (VTE).

**Fig. 3.**
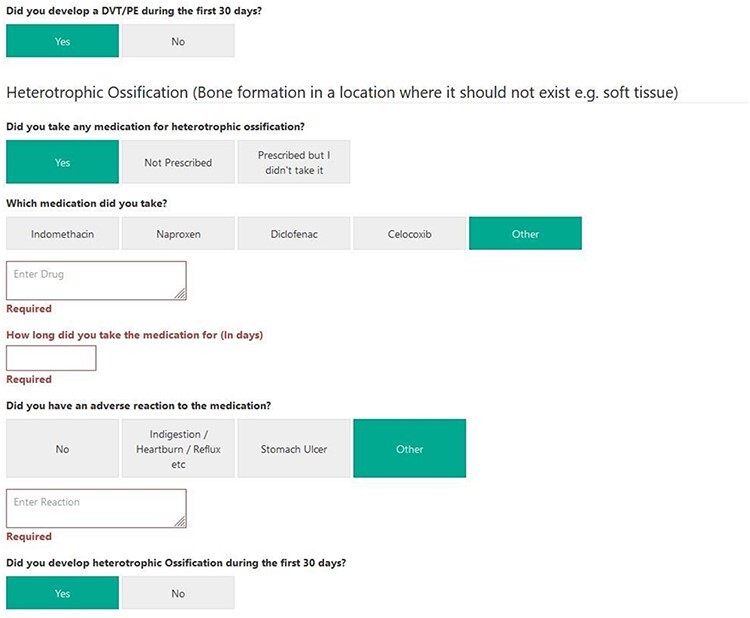
MDS tool for data capture at 30/90 days for VTE and heterotopic ossification.

**Fig. 4.**
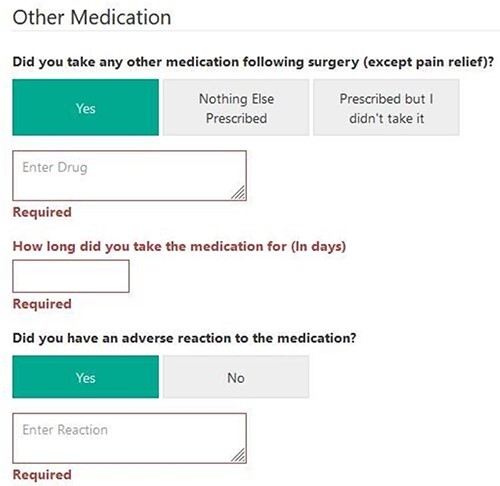
MDS tool for data capture at 30/90 days for other medication.

The MDS form used to collect information was modified, after the approval of the study by the NAHR Board, to reflect this data collection. Surgeons involved in the study were asked to complete an additional section in the MDS and any medication that they prescribed for the patient for the above indications. This automatically triggered a pathway, which sent the patients questionnaires [22] at 30 and 90 days about the extent to which they complied with the regimen and whether they developed any adverse events, or, whether they developed the condition, for which prophylaxis was prescribed. They were also asked about the use of other medications (Figs 2, 3  and 4). They were subsequently asked further questions to assess the efficacy and safety of these medications at other time points. Reminders were sent to the patients if they did not complete the forms. In the pilot centre, further support was provided to assist in data collection locally.

Our outcome of interest was whether the patients completed these forms at 30 and 90 days and whether the local support and administrative support provided for the study improved this compliance and comparison between the pilot centres and non-pilot centres.

The primary aim was to assess the ability of the NAHR to collect high-quality data required for research trials, by measuring compliance with follow-up data collection at pilot centres and comparing this with literature on acceptable follow-up rates, within the scientific literature on clinical trials methodology.

The secondary research aim was to consider quality improvement in registry data collection, assessing whether additional funding and support are needed for data collection when compared with ‘unsupported data collection’. The study included six centres (referred to as pilot centres and known to be performing high volumes of joint preserving surgery), and whilst all centres had access to the new MDS and were informed of its update, the pilot centres received additional support by means of (i) funding to support internal resources to collect data, (ii) additional support from the NAHR registry staff and (iii) additional support from a research fellow, overseeing the project. The control group were non-pilot centres, with other centres contributing to the NAHR and utilizing the same data collection means; however, these were unsupported when considering the above support strategies. The authors hypothesized that additional support would result in an improvement in follow-up rates.

### Ethical considerations

As there was no deviation from routine clinical practice and that this was not a clinical intervention study, there was no additional risk inferred to the patient out of that of usual best care; therefore, it equated to a quality improvement project and not interventional research. Appropriate consent for data collection was sought (a further addition to the MDS). Patients were free to withdraw consent from the NAHR at any stage.

### Study period

The study period was from 9 March 2022 to 9 June 20222. Patients were sent follow-up questionnaires in their post-operative period regarding the outcome measures at 30 and 90 days. The study period to allow 90-day follow-up equated to 7 September 2022, but the data collection was extended until 14 October 2022 so patients could get enough opportunities to complete the questionnaires.

### Statistical analysis

For analysing categorical subgroups, chi-squared test was used to assess between group differences. As this is a non-interventional and non-therapeutic pilot study, ‘effect size’ was not calculated and no power calculation was performed.

## RESULTS

There were 228 patients during the study, 124 of these were amongst the pilot centres and 114 at non-study centres.

During the study period, there were data for eight surgeons amongst the six centres.

### Pilot centres

Follow-up compliance and relative contribution for each centre can be seen in Table I. Compliance of follow-up for all centres was 79% and 69.4% at 30 and 90 days, respectively.

**Table I.** Follow-up and patient contribution during study period

*Study centre*	*Follow-up point*	*Total*	*Completed*	*Percentage complete*
Centre 1	30 days	37	36	97.30
	90 days		36	97.30
Centre 2	30 days	27	22	81.48
	90 days		16	59.26
Centre 3	30 days	27	13	48.15
	90 days		7	25.93
Centre 4	30 days	17	13	75.47
	90 days		12	70.59
Centre 5	30 days	8	8	100
	90 days		8	100
Centre 6	30 days	8	7	87.50
	90 days		7	87.50

**Fig. 5.**
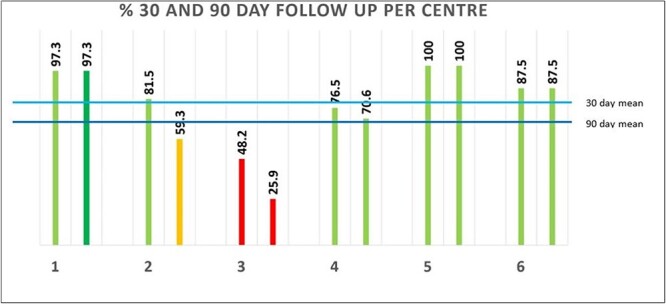
Percentage follow-up rates from each pilot centre.

Follow-up compliance and relative contribution for each pilot centre can be seen in Table I and Fig. 5.

Compliance with follow-up for all centres was 79% and 69.4%. Centres 1, 5 and 6 had a dedicated outcomes coordinator to collect the data.

### Pilot centres—considering Centre 3 as a potential outlier

**Fig. 6.**
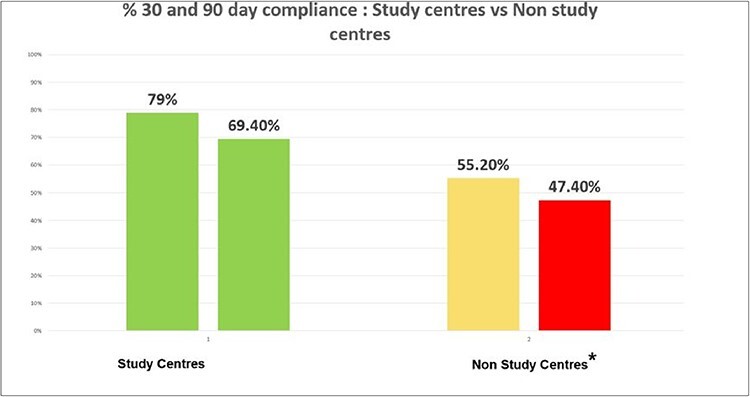
Mean follow-up compliance between study centres and non-study centres.

Noting the discrepancy with Centre 3, sub-analysis was undertaken with results for all centres and with Centre 3 removed. This resulted in a mean follow-up rate, without Centre 3, of 88.7% and 81.4% (Fig. 6).

Whilst the difference at 30 days tended towards significance (*P* = 0.07), it was not statistically significantly different. However, at 90 days, the result was statistically significant (*P* = 0.03).

### Pilot centres versus non-pilot centres

Comparison of compliance for follow-up was undertaken between study pilot centres and non-pilot study centres (Fig. 2) to consider the impact of additional funding and support.

As discussed; follow-up for pilot centres was 79.03% (30 days) and 69.40% (90 days), where as at non-pilot centres, 63 out of 114 patients were followed-up (30 days) and 54 out of 114 (90 days), giving percentage follow-up of 55.20% and 47.40%. The difference in follow-up between centres was statistically significantly different at 30 (*P* = 0.0009) and 90 days.

### Subcategorization and analysis of non-pilot centres

Non-pilot centres were subcategorized based on whether there was an NAHR committee member within the centre or not. The number of patients and follow-up rates were then considered for each group.

Out of the 114 patients, there were 57 in the non-committee centres and 57 in committee centres.

Follow-up for non-committee centres was 24/57 (30 days) (42.11%) and 20/57 (90 days) (35.09%) and for committee centres 39/57 (30 days) (68.42%) and 34/57 (90 days) (59.65%). This difference was statistically significant at both 30 (*P* = 0.0047) and 90 days (*P* = 0.0086).

### Surgeon information

The total number of surgeons during the study period was 34, and a breakdown of their affiliations can be seen in Table II. During the study, 76% of patients were submitted by surgeons with an NAHR affiliation.

**Table II.** Number of surgeons and their contribution during the study period

*Surgeon’s affiliation*	*Surgeon numbers*	*Percentage contribution of data during study period*	*Percentage contribution comparison by NAHR affiliation*
Pilot centre and NAHR committee member	8	52	76
NAHR committee member (non-pilot centre)	3	24	
Non-pilot/non-committee	33	24	24

## DISCUSSION

There are a number of learning points from this study. However, crucially, the authors believe that it is possible to use the registry to collect data on clinical trials prospectively, when considering the follow-up compliance achieved at the pilot, or ‘study-centres’ with the exclusion of Centre 3, which had significant administrative problems, which was 88.7% at 30 days and 81.4% at 90 days. This represented a loss to follow-up of <20% at both time points. This is considered within the acceptable rate of loss to follow-up; however, there may be a potential impact on the validity of results if this were to occur in a randomized control trial.

From a methodological perspective, an improved follow-up rate should be sought in comparison studies; however, as a pilot study from which lessons can be learned, this represents an acceptable rate when considering validity [2] and an improvement when compared with patient-reported outcome measures follow up for total hip arthroplasty and knee arthroplasty in the UK, with a follow-up of ∼40% [23]. Furthermore, three of the six centres achieve a follow-up rate of 87.5% to 100%, which if emulated in a clinical interventional study, would be sufficiently satisfactory to draw conclusions [24]. Interestingly, these centres had a dedicated outcomes team that would assist in data collection.

Considering the results and subsequent statistical analysis, one of the crucial lessons to learn from this study is to not merely select ‘study centres’ solely based on their volume, which would seem intuitive for overall numbers but may yield poor follow-up. From this study, it is apparent that several, alternative, factors should be considered; firstly, the robustness of data management infrastructure and patient administration system within the hospital should be satisfactory. Secondly, the track record of a centre for collecting data for the registry, ensuring satisfactory compliance historically, can be considered a reasonable predictor of future performance.

As discussed in the Results section, there were deficiencies in Centre 3 in the area of hospital administration and the joining of patient administration systems with clinical systems, making it difficult to follow up patients. There were frequent instances of missing patient details or purging of data at certain points, prior to entry on the registry. This was what led to their poor follow-up rate.

Despite the difference seen between ‘pilot centres’ and ‘non-pilot centres, when sub-analysis was undertaken one of the notable variables was, that a small number of ‘non-study centres’ contained a surgeon who was on the NAHR committee. These are senior surgeons and have been involved with the registry since its inception. A statistical analysis of ‘non-study centres’ where they were divided into ‘NAHR committee centre’ and ‘non-NAHR committee centre’ showed a statistically significant difference in follow-up rates (30 days *P* = 0.0047/90 days *P* = 0.0086), highlighting that ‘NAHR committee centres’ had elevated the mean value for ‘non-study centres’. From this study, however, we can see that ‘pilot centres’ achieved better follow-up than ‘non-pilot centres’, suggesting that increased funding and data collection support may have a positive impact on data collection rates, suggesting that irrespective of the role that motivation from NAHR committee surgeons plays, funding and additional support from the registry and the dedicated oversight that occurred in the study improved the results. Therefore, a combination of funding and correct centre selection is most likely to lead to optimal results in an interventional study.

Moving forward, it may be helpful to consider mandatory data submission to the registry to improve outcome rates and monitor outcomes, particularly as it is also recognized that low volume surgery is associated with higher rates of complications, especially where complex cases are undertaken [25–27]. Mandatory submission has been introduced for the National Joint Registry within the UK [28].Once the use of the registry was mandated, compliance rates significantly improved [29].The use of best practice tariffs has also had a positive impact on data collection and in patient in the National Hip Fracture Database a database registry of all hip fractures in the UK. Evidence has shown that where the best practice tariff criteria are met, patients appear to have improved outcomes [30]. Therefore, mandating the registry as well as considering financial incentives may allow for improved compliance rates. Given the positive impact that financial incentivization had on the results of this study, linking reporting to funding may be of benefit.

There are limitations to this study. One of the main issues pertains to the unknown with respect to follow-up. The follow-up seen here is ‘best case’ as there may be a number of patients never entered, who would not have been followed up. This means that there is also the potential for patient selection bias based on the likelihood of the patient engaging with follow-up. Secondarily, to apply the findings of this study to the entire UK would require a significant upscaling of resources to provide the support that would be required.

When considering the existing literature on RRCTs, it has been stated that ‘these trials have less stringent inclusion/exclusion criteria and patient monitoring and follow-up are more akin to the real world than the more intensive monitoring in explanatory trials, which enhances the generalizability of their findings’ [31]. The authors propose that as well as being comparable to current RCTs, they may have their own advantages. However, to ensure that data is valid and verified, the data that centres are entering should undergo an independent and periodic audit and evaluation [31]. This again would require funding, and therefore, mandating the registry and linking it to funding, as aforementioned in other registries, to cover running costs seems rational.

## CONCLUSION

Considering the follow-up compliance here within and responding to the findings of the pilot, the authors propose that the NAHR and other similar registries can be effectively utilised for RRCTs. However, if we are to do so, it is crucial that we learn from the lessons from the pilot study and that if the NAHR can utilize these findings, it could potentially pioneer as the first orthopaedic registry globally to undertake a prospective RRCT.

## Data Availability

Raw data were generated via Amplitude, which manages data for the UK-NAHR. Derived data supporting the findings of this study are available from the corresponding author, if required.
